# Endocytic Uptake, Transport and Macromolecular Interactions of Anionic PAMAM Dendrimers within Lung Tissue

**DOI:** 10.1007/s11095-017-2190-7

**Published:** 2017-06-14

**Authors:** Christopher J. Morris, Ghaith Aljayyoussi, Omar Mansour, Peter Griffiths, Mark Gumbleton

**Affiliations:** 1 School of Pharmacy, University of East Anglia, Norwich Research Park, NR4 7TJ UK; 2 Cardiff School of Pharmacy & Pharmaceutical Sciences, Redwood Building, Cardiff, CF10 3NB UK; 3 Department of Pharmaceutical, Chemical and Environmental Science, University of Greenwich, Medway Campus, Kent, ME4 4TB UK

**Keywords:** dendrimer, endocytosis, lung, polymer, scattering, transport, uptake

## Abstract

**Purpose:**

Polyamidoamine (PAMAM) dendrimers are a promising class of nanocarrier with applications in both small and large molecule drug delivery. Here we report a comprehensive evaluation of the uptake and transport pathways that contribute to the lung disposition of dendrimers.

**Methods:**

Anionic PAMAM dendrimers and control dextran probes were applied to an isolated perfused rat lung (IPRL) model and lung epithelial monolayers. Endocytosis pathways were examined in primary alveolar epithelial cultures by confocal microscopy. Molecular interactions of dendrimers with protein and lipid lung fluid components were studied using small angle neutron scattering (SANS).

**Results:**

Dendrimers were absorbed across the intact lung *via* a passive, size-dependent transport pathway at rates slower than dextrans of similar molecular sizes. SANS investigations of concentration-dependent PAMAM transport in the IPRL confirmed no aggregation of PAMAMs with either albumin or dipalmitoylphosphatidylcholine lung lining fluid components. Distinct endocytic compartments were identified within primary alveolar epithelial cells and their functionality in the rapid uptake of fluorescent dendrimers and model macromolecular probes was confirmed by co-localisation studies.

**Conclusions:**

PAMAM dendrimers display favourable lung biocompatibility but modest lung to blood absorption kinetics. These data support the investigation of dendrimer-based carriers for controlled-release drug delivery to the deep lung.

**Electronic supplementary material:**

The online version of this article (doi:10.1007/s11095-017-2190-7) contains supplementary material, which is available to authorized users.

## Introduction

**Scheme 1:**
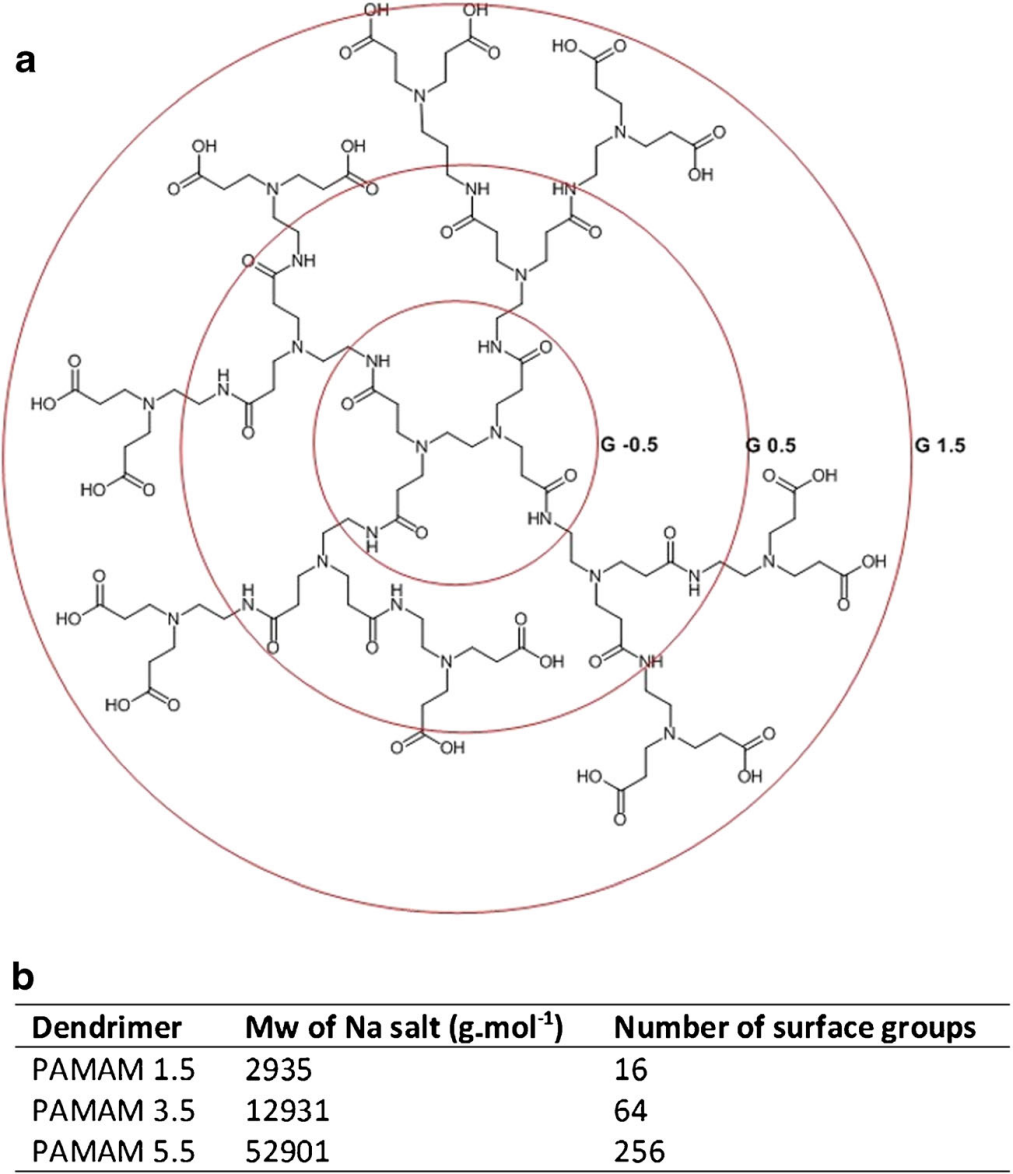
(**a**) Structure of exemplar anionic PAMAM generation 1.5. Iterative branching of each generation is highlighted by concentric circles. (**b**) Molecular mass (Mw) and the number of surface functional groups for the PAMAM generations tested here.

Dendrimers represent an important uniform and nanosized polymer architecture for biomedical and clinical applications and display a variety of synthetic approaches, chemistries, and an ability to carry cargo either by encapsulation, complexation or as pendant groups on the polymer surface. Dendrimers retain promise as nanosized drug carriers displaying a narrow polydispersity and an ease of control in the modification of surface functional groups. The first full family of dendrimer molecules, spanning a number of growth generations were the poly(amidoamine) (PAMAM) dendrimers synthesised by the divergent approach with the iterative addition of methyl acrylate and ethylenediamine to a polymer core and modified with hydroxyl (neutral), amine (positive) or carboxyl (negative) surface functionalities (Scheme 1).

The polyamidoamine (PAMAM) class of dendrimers have been the most widely studied as carriers for low molecular weight drugs, for MRI contrast agents, and as carriers of biomacromolecules including vaccines, peptides, antibodies and DNA. A number of groups have used cationic PAMAM dendrimers to enhance drug delivery across cell membranes, including avoidance of efflux transporters (1), or for example enhancing cytoplasmic delivery of nucleic acid therapeutics (2) or vaccines (3). However, cell membrane disruption and compromise of tight junction integrity is a significant concern for the exploitation of cationic dendrimers, although approaches have been developed to mitigate the biocompatibility issues associated with the use of these platforms (4).

The dendrimer-enhanced delivery of drugs with low oral bioavailability has been of particular interest with many studies (5) reporting dendrimer permeation of cultured intestinal epithelial cell monolayers, although fewer investigations of transport in the fully intact *ex vivo* or *in vivo* tissue have been evidenced. Using an everted gut sac model Wiwatanapatapee *et al*. (6) reported the effect of dendrimer generation and surface functionality (anionic and cationic) upon the association of PAMAM dendrimers with intestinal tissue and upon dendrimer mucosal to serosal transport. In an in-vivo rat model Florence and co-workers (7) administered by oral solution gavage a poly(lysine)_15_ dendrimer bearing at the surface covalently linked C_12_ alkyl chains and studied the subsequent intestinal and whole body tissue accumulation.

In contrast to the oral route, the pulmonary administration of dendrimers has received relatively little consideration and may offer a number of distinct benefits for the localised airway delivery of drugs that require controlled luminal release, but also for the transmucosal or systemic delivery of dendrimer bearing therapeutic cargo. Indeed many reports have highlighted the favourable lung bioavailability of biologics in the absence of absorption enhancers (reviewed in (8,9)). Systematic investigations of lung permeability to nano-sized polymers such as PAMAM dendrimers are limited, and particularly those conducted in a model that retains a fully intact pulmonary architecture.

In this study, we utilised an isolated perfused rat lung (IPRL) model to quantify the rate and extent of absorption from the deep lung of a series of anionic PAMAM dendrimers spanning a molecular weight range 3 to 53 kDa. Following airway administration of the dendrimers in the IPRL model, we observed size-dependent pulmonary transport kinetics for three generations (G1.5, G3.5 and G5.5) of anionic PAMAM dendrimers. In the IPRL, the rate of PAMAM transport from the lung lumen to the pulmonary circulation tended to be less than the dextran probes of comparable molecular dimensions. Further, with respect to G3.5 and G5.5 dendrimers we observed dose-dependent absorption from the airways of the IPRL. Small angle neutron scattering (SANS) studies indicated that this dose-dependency was not attributable to interaction of G3.5 or G5.5 with either of the two principal components of lung lining fluid, i.e. albumin and the zwitterionic surfactant, 1,2-dipalmitoyl-sn-glycero-3-phosphocholine (DPPC). Using lung epithelial cell culture models, we also examined the comparative *in-vitro* permeability of the dendrimers as well as their endocytic uptake and intracellular accumulation within distinct vesicular compartments of lung epithelia. In summary, these data indicate that PAMAM dendrimers offer a promising polymeric delivery platform that could be used to deliver drug cargo either into, or indeed, across the deep lung respiratory epithelium.

## Materials & Methods

### Materials

The following were obtained from Sigma-Aldrich (Poole, UK):anionic PAMAM dendrimer generations (PAMAM) 1.5, PAMAM 3.5 and PAMAM 5.5 (nominal MWs 3KDa, 13KDa, 53KDa, respectively), N-hydroxysulfosuccinimide (sulfo-NHS), 1-ethyl-3(3-dimethylaminopropyl) carbodiimide hydrochloride (EDC), sodium fluorescein (F-Na) and bovine serum albumin (>98% purity) and FITC-dextans (FDx, where x represents the nominal molecular weight in kDa). All tissue culture plastics were from Corning Costar (Hemel Hempstead, UK). Anti-caveolin-1 and anti-EEA-1 antibodies were from BD Biosciences (UK). Oregon Green 488 (OG) cadaverine, fluorescently labelled probes (10 kDa dextran, bovine serum albumin and cholera toxin B subunit) were all from Invitrogen (Paisley, UK). All other chemicals and reagents were of the highest possible quality from Fisher Scientific (Loughborough, UK) or Sigma (Poole, UK). Hydrogenous and deuterated 1,2-dipalmitoyl-*sn*-glycero-3-phosphocholine (DPPC) were from Avanti Polar Lipids Inc. (USA). Sources of other exceptional items are mentioned in the text.

### Fluorescent Labelling of PAMAM Dendrimers

To allow quantification of pulmonary transport and cell uptake, dendrimers were labelled with Oregon Green 488 cadaverine (OG) using carbodiimide-mediated coupling chemistry and purified and characterised as previously described (10). The extent of dendrimer-fluorophore labelling was minimised to reduce interference with the surface properties. Substitution ranged from 1:1.04 to 1:1.31 molar ratio (dendrimer: fluorophore).

### Primary Alveolar Epithelial Cell Cultures

Isolation and primary culture of type II rat alveolar epithelial (ATII) cells to an alveolar epithelial type I cell (ATI-like) phenotype was undertaken according to procedures previously reported (11). All animal experiments and the protocols described below adhered to the Animal (Scientific Procedures) Act 1986 (United Kingdom).

### Permeability of Polarised Calu-3 Monolayers to PAMAM Dendrimers and FITC-Dextrans

Calu-3 monolayers were grown on Transwell® Clear inserts (0.4 μm pore size) for 10–14 days until the transepithelial electrical resistance (TEER) values were >500 Ω.cm^2^ as measured using ENDohm chambers and an EVOM epithelial voltohmeter (Word Precision instruments, Sarosota, USA). Culture media was aspirated, apical and basal chambers were gently washed with warm PBS and bathing fluid replaced with DMEM–F12 (without phenol red). After equilibration (30 min, 37°C) 0.2 mL of dose solution (8 μM or 40 μM PAMAM-OG in serum-free media) was applied to the donor chamber. Cumulative PAMAM-OG transport across the monolayers (37°C, upon an orbital shaker) was measured at pre-determined intervals by sampling 0.2 mL from the basal chamber and replacement with an equal volume of warm media. In addition, transport studies were performed using a molecular weight range of FITC-dextrans and F-Na (40 μM donor concentration) as reference solutes. The cumulative transport of each probe was calculated and an apparent permeability coefficient (P_app_) (cm.s^−1^) determined according to:

1 $$ {P}_{app}=\frac{dM}{dt}\cdot \frac{1}{A.{C}_0} $$

where: δM/δt is the rate of probe accumulation in the basal chamber (calculated from the initial linear portion of the transport curve); A denotes the 0.332 cm^2^ surface area of the Transwell® inserts and C_0_ denotes the initial concentration of probe in the donor chamber.

### Imaging of Endocytic Compartments in Primary Rat Alveolar Epithelial and Calu-3 Cells

Coverslip mounted ATI-like cells were fixed with 3% paraformaldehyde, quenched with 50 mM NH_4_Cl, then permeabilised with 0.2% Triton X-100 in PBS. Coverslips were incubated with primary antibodies against Caveolin-1 (Cav-1), early-endosomal antigen-1 (EEA-1) diluted in blocking buffer (dilutions: Cav-1 1:200; EEA-1 1:200 for 30 min). Lysosomes were labelled by pulse-chase labelling (2 h pulse-3 h chase) with 0.25 mg/mL tetramethylrhodamine-dextran in serum-free medium.

PAMAM-OG uptake was studied in ATI-like cells seeded onto glass bottomed 35 mm culture dishes (MatTek Corporation, Ashland, USA). ATII primary cell were cultured over 6–8 days to achieve an ATI-like phenotype. To assess probe uptake, cells were washed with warm PBS (x3), before addition of PAMAM-OG conjugate (50 nM in DMEM without phenol red, containing 15 mM HEPES) for pre-determined times. Co-internalisation of PAMAM-OG conjugates with 100 μg/mL tetramethylrhodamine-BSA (30 min) or dextran in ATI-like cells was used to co-localise to albumin positive endosomes and lysosomal compartments, respectively. Following incubation at 37°C, cells were washed with pre-warmed PBS then immediately imaged by fluorescent imaging on a Leica DMIRB fluorescent microscope equipped with a 40x objective and attached to a Digital CCD Retiga 1300 camera, processed using Improvision software. Images were cropped and annotated for publication using Photoshop CS2 (Adobe Systems Inc.).

### Pulmonary Translocation of Solutes in an Ex-Vivo IPRL Model

An IPRL preparation using a pMDI intra-tracheal solution instillation technique was used as previously described (12). A critical appraisal of the IPRL model as well as a schematic representation has previously been published (13). PAMAM-OG conjugates were administered to the IPRL in 0.1 mL 20 mM PBS, pH 7.4. Serial aliquots were sampled from the recirculating perfusate at pre-determined times and centrifuged (13,000 *g*, 10 min, 4°C) to remove trace blood cells and the supernatant stored at −80°C prior to quantification by microplate fluorescence spectroscopy (λex 485 nm λem 520 nm; FLUOstar, BMG, Aylesbury, UK). A calibration curve for each PAMAM-OG conjugate was prepared in control perfusate that had recycled through the lung for 60 min. Cumulative transfer of dendrimer into the perfusate was calculated by multiplying the cumulative concentration by the total perfusate volume (~75 mL). The wet:dry ratio of lung tissue was calculated by comparison of the tissue weight immediately after the IPRL experiment (wet weight) and the tissue weight after lyophilisation for 72 h (dry weight). Control experiments involved instillation of PBS dose solution followed by perfusion and ventilation for 60 min.

### Lipid Vesicle Preparation

Small unilamellar vesicles comprising h-DPPC or d-DPPC were prepared using the freeze/thaw extrusion method. DPPC was dissolved in chloroform (10 mg /mL) in a round bottom flask then solvent was removed by rotary evaporation to yield a phospholipid film. Residual solvent was removed under high vacuum for 1 h. The film was rehydrated in 2 mL deuterated phosphate-buffered saline pH 6.5 (Sigma-Aldrich, Poole, UK) then incubated at 50°C for 1 h. The hydrated film was then subjected to five freeze/thaw cycles which involved freezing for 5 min in liquid nitrogen followed by thawing for 5 min in water at 50°C and then vortex mixing for 5 min. Finally, the lipid mixture was extruded under high pressure, firstly through a 200 nm polycarbonate membrane (10 times) and secondly through a 100 nm polycarbonate membrane (20 times) using The Extruder (Lipex Biomembranes Inc., Vancouver, Canada) maintained at 50°C.

### SANS Studies on PAMAM Dendrimer in Lung Fluid Models

SANS experiments were performed on the D11 diffractometer based at the steady-state reactor source at the Institut Laue-Langevin (ILL) in Grenoble, France. A *Q* range of 0.01 Å^−1^ to 0.5 Å^−1^ was selected by choosing several instrument settings (sample-to-detector distances, collimation) with a constant neutron wavelength (*λ*) of 8 Å, where:

2 $$ Q=\left(4\pi /\lambda \right) \sin \left(\vartheta /2\right)\mathrm{Q}=\left(\frac{4\uppi}{\uplambda}\right) \sin \left(\frac{\uptheta}{2}\right) $$

Samples were contained in 1 mm pathlength, UV-spectrophotometer grade, quartz cuvettes (Hellma) and thermostatically controlled at 25 ± 0.5°C can be achieved. Experimental measuring times were dependent on the sample-detector distance, but approximately 40–60 min overall.

All scattering data were normalised for the sample transmission and the incident wavelength distribution, corrected for instrumental and sample backgrounds using a quartz cell filled with D_2_O, and corrected for the linearity and efficiency of the detector response using the instrument specific software package. The data were put onto an absolute scale using a well-characterized partially deuterated polystyrene blend standard sample.

Previous SANS studies on PAMAMs have shown that they form spherical structures (14). Here, the data were fitted to a model that describes the scatterer as a solid, spherical object. In this representation, the scattered intensity, I(Q), is expressed as:

3 $$ I(Q)=\frac{scale}{\mathrm{v}}.{\left[3 V\left(\varDelta \rho \right).\frac{\mathit{\sin}(qr)- qr\ \mathit{\cos}(qr)}{(qr)^3}\right]}^2+{B}_{inc}\kern1.25em $$

Where *scale* is a volume fraction, *V* is the volume of the scatterer, *r* is the radius of the sphere, *∆ρ* is the difference between the scattering length density of the scatterer and the solvent, and *B*_*inc*_ is the incoherent background scattering. The data were mathematically analysed using the open source software, SasView (15).

A number of mathematical models have been explored in this work, the one that warrants discussion here being the unilamellar vesicle one applicable to the scattering from DPPC based systems. In this formulism, the shape of the vesicle is modelled as a series of concentric spheres of varying radii (outermost corona – hydrated phospholipid headgroups; middle corona – hydrocarbon region, with thickness typically twice the length of the hydrocarbon tail; innermost corona - hydrated phospholipid headgroups):

4 $$ P(Q)=\frac{\varnothing }{V_{shell}}{\left[\frac{3{V}_{core}\left({\rho}_{solvent}-{\rho}_{shell}\right){j}_1\left( q{R}_{core\ }\right)}{q{R}_{core}}+\frac{3{V}_{tot}\left({\rho}_{shell}-{\rho}_{solvent}\right){j}_1\left( q{R}_{tot}\right)}{q{R}_{tot}}\right]}^2+{B}_{inc} $$

where ∅ is the volume fraction of shell material, V_shell_ is the volume of the shell, V_core_ is the volume of the core, V_tot_ is the total volume, R_core_ is the radius of the core, R_tot_ is the outer radius of the shell, ρ_solvent_ is the SLD of the solvent (similar to the SLD of the core in this model), ρ_shell_ is the SLD of the shell and j_1_ is the spherical Bessel function. In this study, scattering from the phospholipids has been modulated by employing tail-deuterated DPPC, which shows a significantly weakened scattering as the hydrocarbon corona is now largely invisible against a D_2_O background (16).

### Statistical / Data Analysis

Non-linear regression analysis of the initial transport kinetics of PAMAM-OG across the IPL (0–60 min) was performed using GraphPad Prism (v5.01) according to Eq. 5 with the recycling perfusate represented as a single accumulating compartment receiving first-order initial input (K_*ini*_).

5 $$ M= D. F\left(1-{e}^{k_{ini}. t}\right) $$

Where M is the cumulative mass of probe transported across the lung into the perfusate (0–60 min); D is the nominal dose of PAMAM conjugate, F is the fraction of the dose deposited in the lung, correcting for losses in the dosing apparatus, K_*ini*_ is the rate constant (min^−1^).

For treatment comparisons results are reported ± S.D. One-way analysis of variance (ANOVA) was used to compare cumulative absorption data. *Post-hoc* analysis was performed using Newman-Keuls Multiple comparison. Statistical significance was determined at *p* < 0.05.

## Results

### *In-Vitro* Transport of PAMAM and Dextran Probes

**Fig. 1:**
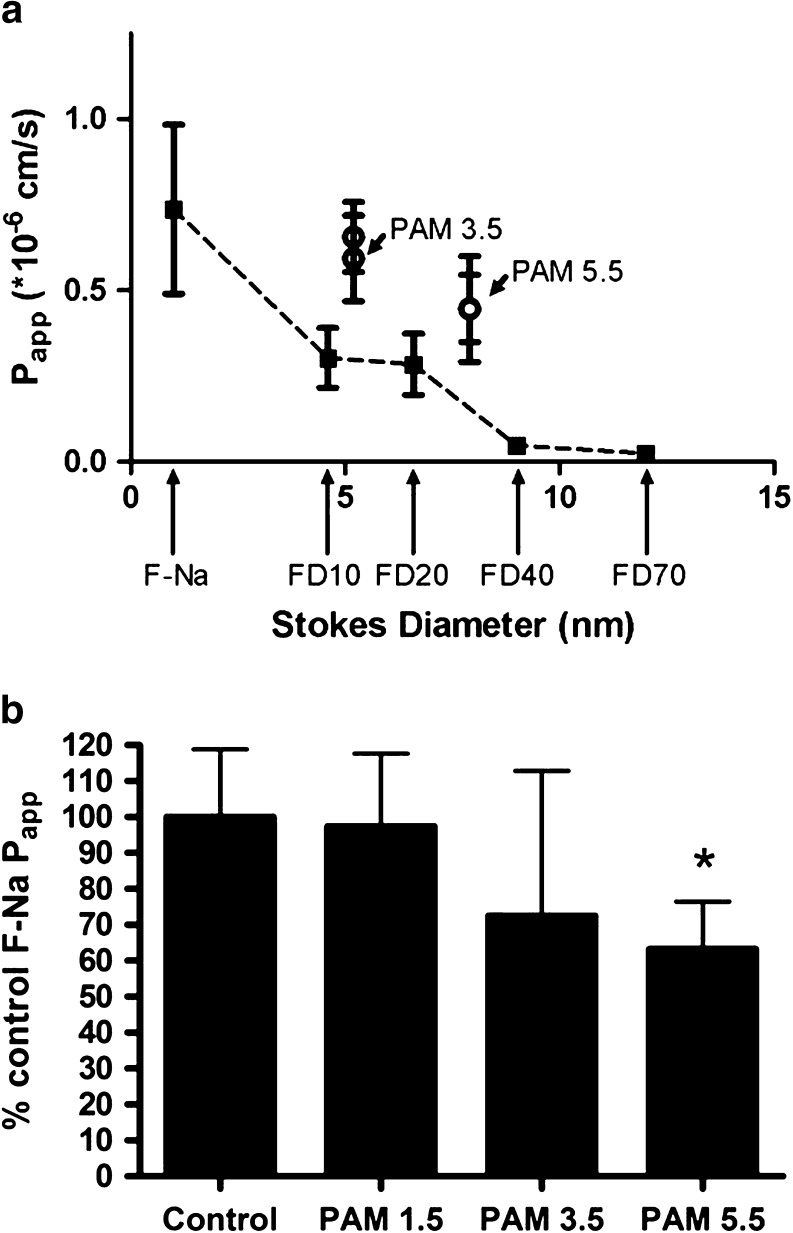
*In-vitro* transport of dendrimers and dextran probes in lung epithelial cells (**1a**) Permeability of *in-vitro* Calu-3 cell monolayers showing a plot of P_app_ (x 10^−6^ cm/s) as a function of Stokes diameter, and demonstrating molecular size-dependent transport of dextrans (FD), with an overlay of F-Na permeability. The permeability of the dendrimers, PAMAM3.5 and PAMAM5.5, being considerably greater than expected based on molecular size alone; (**1b**) Relative permeability of the paracellular probe F-Na and the impact of co-incubation with unlabelled PAMAM1.5, PAMAM3.5 and PAMAM5.5. All data *n* = 4–6 ± standard deviation. * denotes *p* < 0.05 compared to control by one-way ANOVA and Newman-Keuls multiple comparison test.

We firstly examined the transepithelial transport of anionic PAMAM dendrimers across the Calu-3 bronchial cell line, and compared the transport to that of a range of fluorescein-labelled dextrans (FD) whose molecular size spanned that of the dendrimers. Scheme 1 includes the generic molecular structure of low generation number anionic PAMAM dendrimers, as well as the molecular masses and the number of surface functional groups of PAMAMs examined herein. Calu-3 cell monolayers were cultured until they reached a TEER of >500 Ω.cm^2^, typically around day 10–15 post-seeding. The apparent permeability coefficients (P_app_) determined from the initial linear transport rates are shown in Table I. The P_app_ for both PAMAM3.5 and PAMAM5.5 were dose-independent with, for example, the P_app_ for PAMAM3.5 at 40 μM and 8 μM being respectively, 0.656 *vs* 0.593 (x 10^−6^) cm.s^−1^. Therefore, over this concentration range the transport of both PAMAM3.5 and PAMAM5.5 was predominantly one of passive diffusion. Although the P_app_ data for the larger molecular sized PAMAM5.5 was consistently lower than that for the PAMAM3.5 this was not statistical significant (*p* > 0.05) despite the difference in molecular weight and molecular diameters reported elsewhere (17). The comparative permeability profile of Calu-3 monolayers to F-Na and a range of FDs, whose Stokes diameters spanned 1.5 to 12 nm (Table I), was also studied. The rate of F-Na and FD transport from a 40 μM dose solution showed a clear inverse relationship to molecular size, with the smallest probe, F-Na permeating most rapidly, P_app_ averaging 0.737 (x 10^−6^) cm.s^−1^, whereas FD-70, the largest solute, permeating some 30 times more slowly with an average P_app_ of 0.023 (x 10^−6^) cm.s^−1^ (Table I). Figure 1a shows such a relationship in a plot of P_app_ against solute molecular diameter for the FD panel (filled squares). The plot also overlays the permeability data for PAMAM3.5 and PAMAM5.5 (empty circles) whose permeabilities are clearly greater than for the dextrans of a molecular size.

**Table I:** Permeability of Calu-3 and Caco-2 Monolayers. Data Shown are Expressed as Mean ± SD (*n* = 4–6). For the 40 μM PAMAM Concentration Data the Symbol *^1^ Indicates Statistical Difference (*p* < 0.05) to the Permeation of All Dextrans (FD10-FD70), While *^2^ Indicates Statistical Difference (*p* < 0.05) to only FD 40 and FD70. The Symbol *^3^ Indicates a Significant Difference (*P* < 0.05) in Permeability Between Caco-2 and Calu-3 Monolayers for the Respective Probe

Probe and cell line	Stokes diameter (nm)	Donor concentration	Apparent permeability coefficient (P_app_) (x 10^−6^ cm.s^−1^)
Calu-3			
PAMAM3.5	5.2	40 μM	0.656 ± 0.102^*1^
PAMAM3.5	5.2	8 μM	0.593 ± 0.125
PAMAM5.5	7.9	40 μM	0.445 ± 0.154 ^*2^
PAMAM5.5	7.9	8 μM	0.447 ± 0.098
F-Na	1.5	40 μM	0.737 ± 0.247 ^*1^
FD10	4.6	40 μM	0.303 ± 0.087
FD20	6.6	40 μM	0.284 ± 0.089
FD40	9	40 μM	0.047 ± 0.016
FD70	12	40 μM	0.023 ± 0.011
Caco-2			
F-Na	1.5	40 μM	0.345 ± 0.039 ^*3^
PAMAM3.5	5.2	40 μM	0.194 ± 0.026 ^*3^

We also undertook comparative transport studies in the Caco-2 model (Table I). As a reference the P_app_ to F-Na was significantly (*p* < 0.05) lower for the Caco-2 monolayers compared to the Calu-3 cells not withstanding TEER values which were comparable ca. 400–500 Ω.cm^2^. Similarly the permeability to PAMAM3.5 was significantly (*p* < 0.05) lower in the Caco-2 cells, with the transport of PAMAM3.5 some 56% (*p* < 0.05) of the F-Na permeability in Caco-2, whereas in Calu-3, the transport of PAMAM3.5 was 89% of that of the F-Na probe (*p* > 0.05).

We next examined if the anionic PAMAM dendrimers themselves could alter paracellular permeability by co-application to the Calu-3 monolayers of unlabelled dendrimers (each at 40 μM) together with F-Na as the paracellular probe. Co-application of PAMAM1.5 caused no significant change (*p* > 0.05) in the permeability to F-Na (Fig. 1b), while co-application of PAMAM3.5 displayed a trend to reduce F-Na transport although not to any significant (*P* > 0.05) difference. In contrast, 40 μM PAMAM5.5 caused a 35% reduction (*p* < 0.05) in the P_app_ of F-Na.

### Endocytic Uptake and Trafficking of PAMAMs in Lung Epithelia

**Fig. 2:**
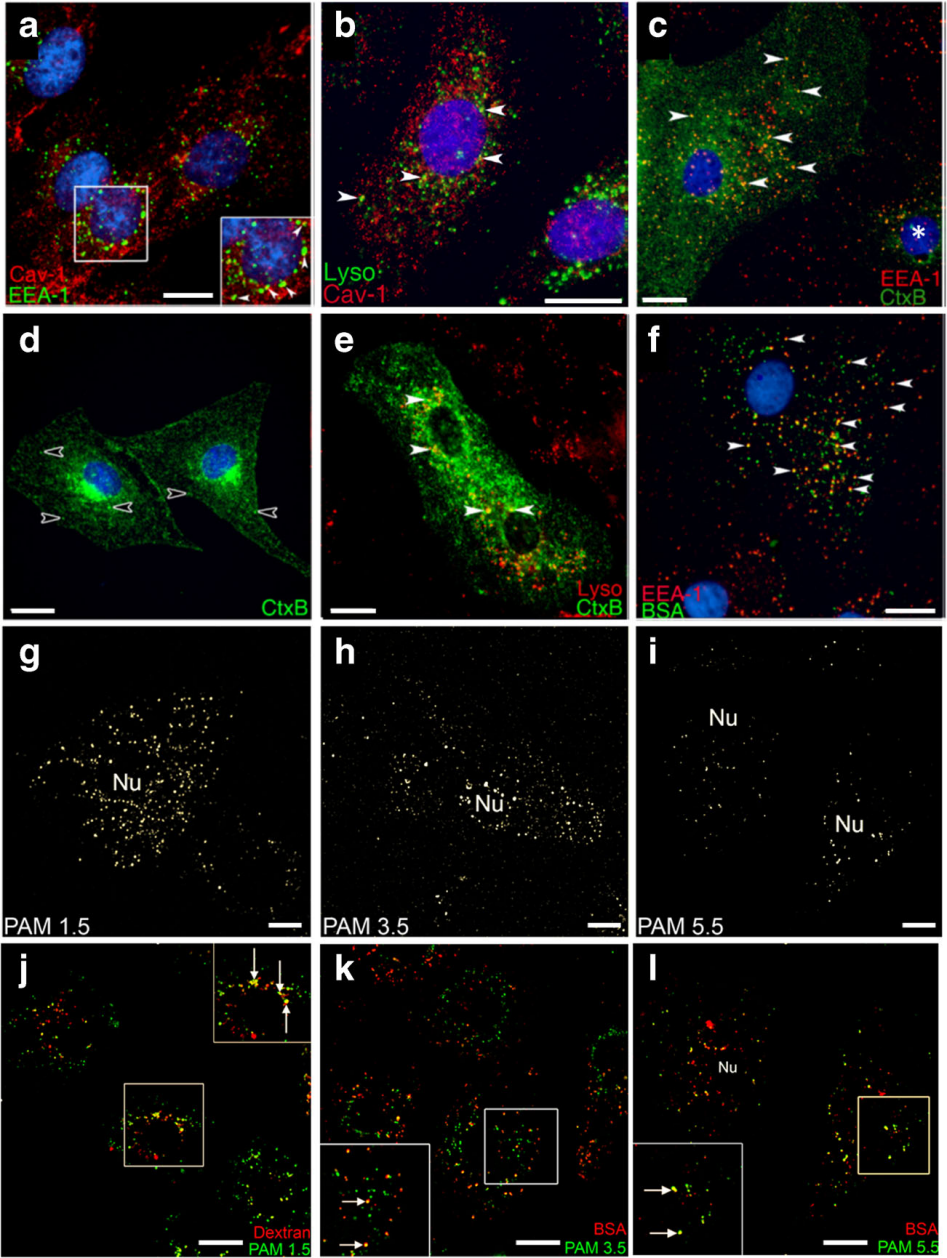
Distribution and functionality of endocytic compartments in ATI-like lung epithelia**.** Co-localisation of Caveolin-1 positive vesicles with (**2a**) EEA-1 immunopositive endosomes and (**2b**) lysosomes labelled by a 2 h/3 h pulse-chase internalization of dextran. CtxB internalized into ATI-like cells localizes to (**2c**) early endosomes after 10 min, (**2d**) a peri-nuclear compartment after 45 min. (**2e**) only a minor fraction of CtxB puncta localized to lysosomes following a 2 h/3 h pulse-chase co-internalisation of CtxB and 10 kDa dextran. Empty arrowheads in 2D highlight non-perinuclear puncta present within upstream compartments. (**2f**) TMR-labelled BSA accumulated in EEA-1 positive endosomes after a 10 min incubation. (**2g–I**) Primary ATI-like cells show a decrease in the density of PAMAM-OG staining density pattern that accompanies an increase in dendrimer generation from 1.5 to 5.5 after 30 min incubation. (**j**) a fraction of PAMAM 1.5 co-localised with dextran labelled lysosomes after a 2 h/3 h pulse-chase co-internalisation. Both PAMAM 3.5 (**k**) and PAMAM 5.5 (**l**) co-localise with BSA after 30 mins co-internalisation. Arrows in zoomed section highlight co-localised puncta. Scale bar: 5 μm.

Figure 2a-b demonstrate a degree of separation between the caveolar membrane system and both the early endosomal compartment labelled with EEA-1 (Fig. 2a, green) and the pulse-chase dextran labelled lysosomal compartment (Fig. 2b, green). As expected, Cav-1 puncta were widely distributed across the ATI-like cell (18). EEA-1 immunostain (Fig. 2a) was most often identified in regions of concentrated puncta in the peri-nuclear region, wherein a small degree of co-localisation was observed (arrows in zoomed insert, Fig. 2a). Similarly, in Fig. 2b it is apparent that the majority of dextran-positive lysosomes (green) did not co-localise with Cav-1 staining (red). To examine the functionality of these endocytic compartments we exposed ATI-like cells to two macromolecular probes – cholera toxin B subunit and albumin – both of which are used as probes of caveolae-dependent endocytosis (19,20). ATI-like cell cultures incubated with CtxB for 10 min (Fig. 2c) displayed a heterogeneous staining pattern. In some cells, a 10 min internalisation period produced widespread green staining (left-hand cell, Fig. 2c) and significant overlap between CtxB puncta and EEA-1 positive early endosomes (arrowheads, Fig. 2c). In contrast, other cells contained a more restricted staining intensity (e.g. right hand cell marked with an asterix in Fig. 2c). This observation is consistent with the heterogeneous expression of the GM_1_ sphingolipid receptor reported within other cell types (21). The speckled CtxB staining pattern persisted after longer incubation periods (arrows, Fig. 2d), however this was accompanied by a polarised accumulation of CtxB signal in the juxta-nuclear region (Fig. 2d). Notably, some CtxB vesicles occupied peripheral locations within the cell (empty arrowheads, Fig. 2d). Co-localisation of the peri-nuclear CtxB staining to the *trans*-Golgi network (TGN) using a polyclonal anti-primate TGN-46 antibody was impossible due to poor cross-reactivity to rat antigens. However, CtxB displayed extensive overlap with the TGN-46 labelled compartment in A549 human alveolar epithelial cells after 60 min internalisation (Fig. S1). The post-Golgi trafficking of CtxB was examined following an extended 2 h/3 h pulse-chase co-internalisation with dextran (Fig. 2e). There was limited evidence of CtxB trafficking to dextran-positive lysosomes in the peri-nuclear region (arrowheads, Fig. 2e). Instead, we observed a reticular CtxB staining pattern, which indicates the retrograde trafficking of CtxB to the endoplasmic reticulum via the TGN (22).

After 10 min albumin (BSA) internalisation into ATI-like cells extensive localisation of fluorescent albumin puncta (green, Fig. 2f) to an EEA-1 positive compartment was observed. In contrast to the CtxB staining pattern, albumin staining was less extensive across the cell volume and more punctate in nature.

A 30 min internalisation of OG-labelled PAMAMs into ATI-like cells resulted in a decreasing intensity of scattered cytoplasmic puncta as PAMAM molecular size increased (Fig. 2g-i). This was not a result of OG-labelling efficiency between the different PAMAM species as the same labelling stoichiometry was applied and confirmed in each case. Uptake of the PAMAM dendrimers into cytoplasmic vesicles was confirmed by co-localisation with endocytic marker probes, fluorescent 10 kDa dextran (red, Fig. 2j) and bovine serum albumin (red, Figs. 2k,l). These probes were specifically chosen for their comparable molecular dimensions to PAMAMs 1.5, 3.5 and 5.5 and well understood endocytic pathways. A 2 h/3 h pulse-chase co-internalisation of PAMAM 1.5 and 10 kDa TMR-dextran (Fig. 2j) showed a fraction of internalised dendrimer co-localised with the dextran within peri-nuclear lysosomal compartments. A proportion of the PAMAM 1.5 puncta remained green, not co-localised and indicating accumulation in upstream endocytic compartments. Co-internalisation of PAMAM 3.5 and PAMAM 5.5 with BSA (Fig. 2k and l, respectively) resulted in both noticeable accumulation of PAMAM puncta in the perinuclear region as well as co-localisation with BSA positive puncta scattered through the cell.

### Transmucosal Transport of PAMAMs in the Intact Lung

**Fig. 3:**
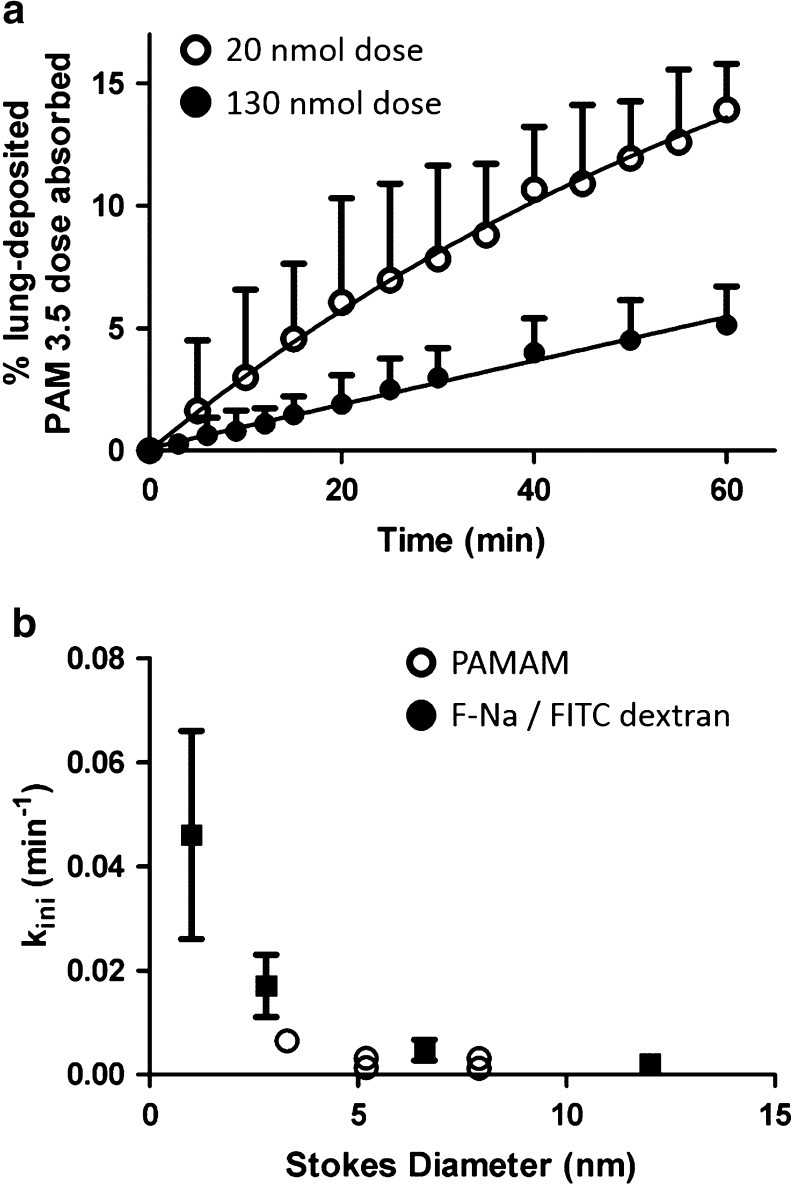
**Pulmonary transport of anionic PAMAM dendrimers across the IPRL.** (**a**) Example cumulative transport profile for the airway to perfusate absorption of PAMAM 3.5 following instillation of 20 nmol (empty circles) or 130 nmol (filled circles); (**b**) shows the relationship between solute Stokes diameter and the k_*ini*_ rate constant for PAMAM dendrimers (empty circles) and FD or F-Na (filled squares). Two empty symbols are shown in panel B when two different PAMAM dose levels were tested. Data shown are mean ± SD, *n* = 3–4.

The cumulative absorptive kinetics of PAMAM 1.5, PAMAM 3.5 and PAMAM 5.5 were determined following pMDI-mediated solution instillation into the distal airways of the IPRL model. Figure 3a shows, as an example, the IPRL absorptive transport profiles for PAMAM 3.5 following either low dose (20 nmol; empty symbols) or high dose (130 nmol; filled symbols) administration; the Figure expresses the absorption data as a % of lung deposited dose, a parameterisation which clearly emphasises the different kinetic outcomes. Specifically, both the transport rate constant (K_*ini*_) and the 60 min extent (%) of PAMAM3.5 dose absorbed were for the low dose more than double that recorded for the high dose (*p* < 0.05, Fig. 3a and Table II). Such dose-dependent kinetics (low dose showing a faster and greater extent of absorption compared to high dose) was also evident for the PAMAM5.5 administration (Table II). At near equivalent mole doses we observed what appeared to be a distinct molecular size division in the absorption of the dendrimer species with the extent and rate constant of absorption for PAMAM1.5 (3.3 nm diameter) markedly greater (*P* < 0.05) than both the PAMAM3.5 (5.2 nm) and PAMAM5.5 (7.9 nm) species. Furthermore we found the kinetics of absorption for the latter two dendrimers species to essentially be indistinguishable (*P* > 0.05) following either the low dose comparison or the high dose comparison (Table II).

**Table II:** Pulmonary Transport Kinetic Parameters for Macromolecular FD and PAMAM Solutes. Data are Mean ± SD, *n* = 3 Except F-Na (40 μg Dose) Where *n* = 14. † Denotes Studies Conducted over 60 min

Solute	PAMAM 1.5	PAMAM 3.5		PAMAM 5.5		F-Na		FD4	FD20		FD70
Nominal lung deposited dose	150 nmol	Low dose 20 nmol	High dose 130 nmol	Low dose 23 nmol	High dose 91 nmol	40 μg 100 nmol	160 μg 400 nmol	200 μg 50 nmol	Low dose 200 μg 10 nmol	High dose 2000 μg 100 nmol	200 μg 4 nmol
% dose absorbed at 60 min	20.5 ± 7.5	13.9 ± 1.9	5.1 ± 1.6^†^	14.5 ± 5.5	8.6 ± 3.4^†^	75.7 ± 1.4	61.9 ± 8.6^††^	42.5 ± 8.2	22.4 ± 7.4	20.7 ± 4.1^††^	8.6 ± 2.5
*K*_*in*_ (min ^−1^ )	0.0065 ± 0.0006	0.0032 ± 0.0004	0.0014 ± 0.0005^†^	0.0031 ± 0.0006	0.0012 ± 0.0002^†^	0.039 ± 0.008	0.046 ± 0.02^††^	0.017 ± 0.006	0.0043 ± 0.001	0.0047 ± 0.0002^††^	0.0021 ± 0.0002
M_w_ (g.mo1^−1^)	2935	12,931		52,901		396		3820	20,200		75,090
Hydrodynamic diameter (nm) ^$^	3.3	5.2		7.9		1.0		2.8	6.6		12

^$^values taken from {Fritzinger *et al.* (17) #67}

^†^indicates a significant difference (*p* > 0.05) between the parameters for the two instilled doses

^††^no difference (*p* > 0.05) between the parameters for the two instilled doses

To provide a comparative permeability profile within the IPRL model we also instilled a range of fluorescently labelled dextrans (FD) whose molecular weights (3.8 kDa to 75.1 kDa) and diameters (2.8 nm to 12 nm) spanned that of the PAMAM dendrimers. For the dextran FD20 (20.2 kDa, 6.6 nm) we tested if dose-dependent absorption kinetics were evident across 10 to 100 nmole dose instillations. In contrast to PAMAM3.5 and 5.5 we found no evidence of a dose-dependent absorption (*P* > 0.05; Table II). We also found the same lack of dose-dependency for the small paracellular probe, sodium fluorescein (F-Na) (Table II). Table II also shows how the IPRL absorptive parameters for the FDs varied as a function of size; incremental decreases in the rate and extent of transport of the FDs within the IPRL evident with increasing molecular weight and size. For example, FD40 was transported x5-fold more extensively and with a rate constant some x8-fold greater than that of FD70 (Table II). Our data are exemplified for the FDs (and F-Na) in Fig. 3b, which also has an overlay of the equivalent data for the PAMAM dendrimers. It is clear that PAMAM dendrimers were somewhat more restricted in their transport than an equivalent sized FD, which may reflect contrasting molecular shape between the dendrimers and the dextran polymers.

Here as part of the IPRL studies we sought to examine dendrimer biocompatibility, albeit at a gross-level. We found no acute evidence of the appearance of oedema, a qualitative observation supported by monitoring lung wet:dry weight ratios. We found no difference (*P* > 0.05) in lung wet:dry weight ratios between control lungs (IPRL run for 60 min following instillation of an equivalent dosing volume of PBS) at 4.91 ± 0.30, and OG-labelled PAMAM1.5 (4.67 ± 0.02), PAMAM3.5 (4.78 ± 1.02) and PAMAM5.5 (4.34 ± 0.25) dosed lungs. Monitoring paracellular permeability of the IPRL with F-Na we observed that co-instillation of PAMAM3.5 and F-Na caused no significant change (*p* > 0.05) in the permeability to F-Na (K_*ini*_, min^−1^ control (*n* = 14) 0.039 ± 0.009 *vs* PAMAM3.5 (*n* = 4) 0.043 ± 0.016). However, F-Na co-instilled with PAMAM5.5 led to a decrease (*P* < 0.05) in the permeability to F-Na (K_*ini*_ min^−1^ control (*n* = 14) 0.039 ± 0.009 *vs* PAMAM 5.5 (*n* = 4) 0.018 ± 0.002).

### SANS Investigations of PAMAM-Macromolecule Interactions

**Fig. 4:**
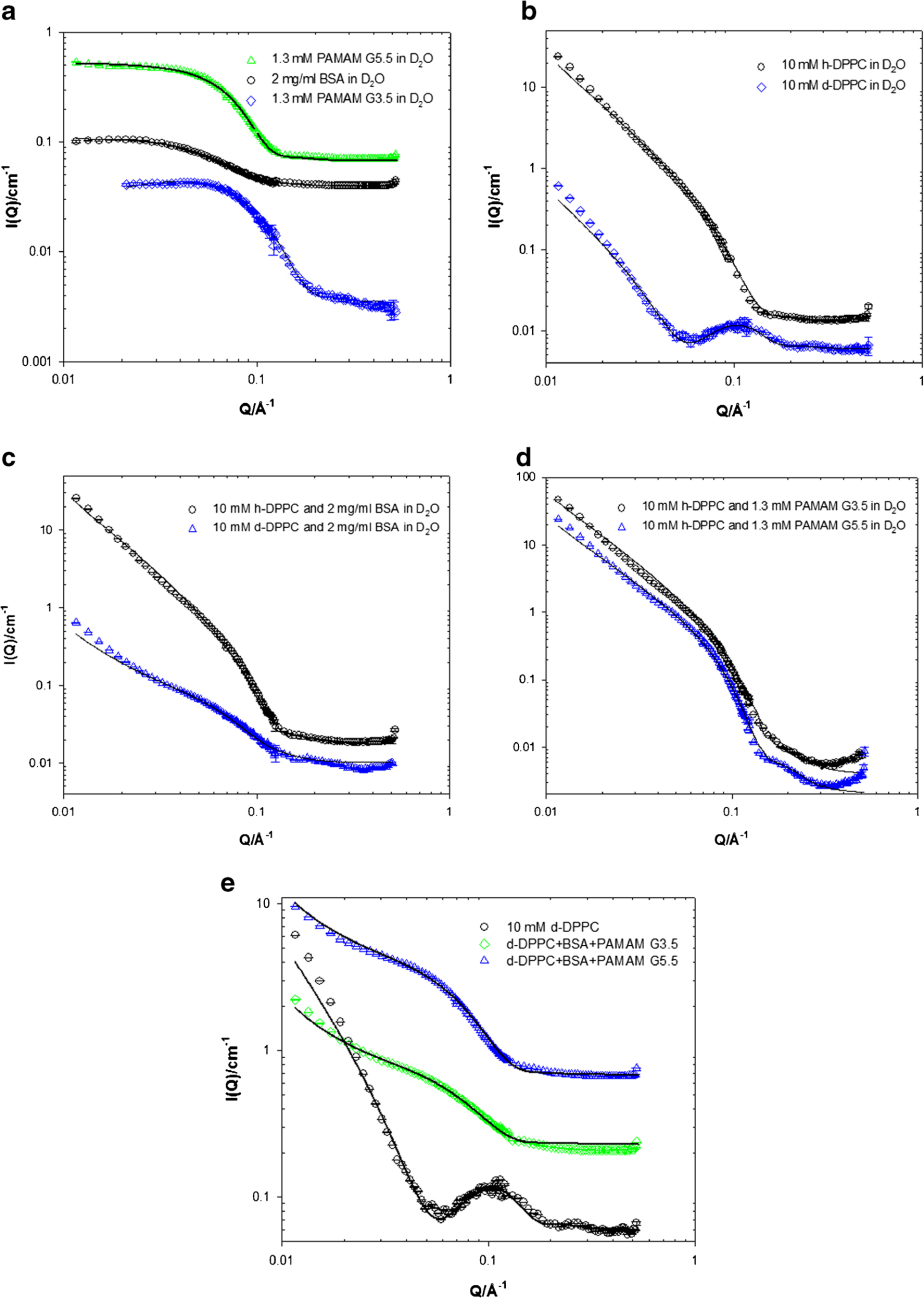
SANS as a function of (**a**) PAMAMs/BSA, (**b**) h and d-DPPC, (**c**) mixtures of h-DPPC/BSA and d-DPPC/BSA, (**d**) mixtures of h-DPPC/PAMAM G3.5 and h-DPPC/PAMAM G5.5, (e) mixtures of 10 mM d-DPPC/4 mg.ml-1 BSA/1.3 mM PAMAM G3.5 and 10 mM d-DPPC/4 mg.ml-1 BSA/1.3 mM PAMAM G5.5. Solid lines corresponds to the model fits as discussed in the text. Data has been offset for clarity.

We next employed small angle neutron scattering (SANS) to investigate the molecular structure of PAMAMs in binary and tertiary models of lung lining fluid. To establish baseline data we modelled the SANS from BSA and PAMAM3.5 and 5.5 in deuterated water (Fig. 4a). The models determined the molecular radii (*R*_*G*_) for BSA, PAMAM 3.5 and PAMAM 5.5 of 28, 20 and 30 Å, respectively. These PAMAM dimensions are consistent with hydrodynamic radii data determined by others using diffusion NMR (17). The use of deuterated DPPC (d-DPPC) in D_2_O reduces the normally high scattering intensity of hydrogen-containing lipids (h-DPPC) in D_2_O. This “contrast” approach was used to determine that the DPPC SUVs displayed radii of 580 Å with a bilayer thickness of 30–40 Å (Fig. 4b, Table III). When we made binary mixtures of DPPC with BSA (Fig. 4c) or with PAMAM 3.5 or 5.5 (Fig. 4d) we found the scattering intensities of binary mixtures were equal to the sum of their components parts, thus indicating no significant interaction between the paired components. Similarly, the scattering intensities of ternary solutions (lipid, PAMAM, protein, Fig. 4e, Table III) were equivalent to spectra predicted by simple addition of scattering intensities from the individual components.

**Table III:** Structural Key Parameters of 10 mM h-DPPC / d-DPPC Obtained from the SANS Model Fitting as a Function of PAMAMs or BSA Concentration at 25°C in D_2_O. ND: Not Determinable Due to Low Contrast

Sample description	Vesicle radius (± 10, Å)	Vesicle bilayer thickness (± 5, Å)
h-DPPC	580	38
h-DPPC +1.3 mM PAMAM G3.5	580	35
h-DPPC +1.3 mM PAMAM G5.5	570	35
h-DPPC +2 mg.ml^−1^ BSA	580	40
d-DPPC	ND	30
d-DPPC +4 mg.ml^−1^ BSA	580	50
d-DPPC +1.3 mM PAMAM G3.5 and 4 mg.ml^−1^ BSA	580	35
d-DPPC +1.3 mM PAMAM G5.5 and 4 mg.ml^−1^ BSA	580	50

## Discussion

In this work we sought to provide a detailed examination of the pulmonary disposition of anionic PAMAM dendrimers using a combination of in vitro cell cultures and intact ex vivo IPRL model. Transport experiments using restrictive, polarised Calu-3 monolayers demonstrated that PAMAM3.5 and PAMAM5.5 permeate these monolayers at rates higher than control solutes (FITC-dextrans) of comparable molecular dimensions. Unexpectedly, the permeability to F-Na decreased when co-applied with PAMAM 5.5. That the monolayer TEER remained stable throughout the studies leads us to interpret this apparent reduced F-Na permeability to reflect a degree of sequestration of F-Na by the dendrimer, and hence a reduced F-Na concentration available for free transport. Nevertheless, together the data supported the conclusion that tight junctional modulation is not a key factor in anionic PAMAM transport across Calu-3.

The interaction between PAMAM dendrimers and epithelial tight junctional proteins has been investigated by a number of groups, principally using the Caco-2 cell model. Avaritt and Swann reported the anionic PAMAM3.5 to lead to a concentration-dependent increase in immunofluorescence staining intensity for tight junction-associated proteins, although this was not associated with an increased protein expression or altered functional paracellular permeability. In contrast, the cationic PAMAM G4 resulted in significant increases in paracellular permeability through a mechanism(s) mediated via intracellular Ca^2+^ release. Two previous studies have reported PAMAM3.5 transport across Caco-2 cells, both studies applying very high dendrimer concentrations (1000 μM) and with markedly different permeability outcomes, i.e. Jevprasanephant *et al*. (23) reporting a P_app_ of 0.02 (x10^−6^) cm.s^−1^ while Kitchens *et al*. (24) reported a 300-fold higher P_app_ of 6 (x10^−6^) cm.s^−1^. Nevertheless, this reinforces the disconnection that can be apparent between different *in-vitro* cellular barrier modelss, the TEER measurements and the permeability of paracellular probes and the transport of experimental agents such the dendrimers; a disconnection that supports studies in a more physiologic lung model that reflects the epithelial barriers of the deep lung.

A number of published reports have concluded that endocytic uptake and / or transcytosis across epithelial barriers could play a significant role in the net transmucosal dendrimer transport (6,25). We next gathered spatiotemporal information on the expression and functionality of macromolecular uptake pathways to examine their role in dendrimer uptake by lung alveolar cells. A combination of fixed cell immunolabelling and live cell imaging of primary rat ATI-like cells confirmed limited co-localisation of intracellular populations of Cav-1 positive, EEA-1 positive and dextran-loaded lysosomes. Spatial segregation is a key feature of polarised epithelial cells such as lung alveolar epithelia because it affords proper control of many key processes including, but not limited to, cell adhesion, migration and nutrient uptake (26). Each of these distinct endocytic compartments was shown to fulfil a functional role in the uptake of probes with molecular dimensions comparable to the size range of PAMAM dendrimers (3.3–7.9 nm) that were studied here i.e. 10 kDa dextran (4.6 nm), CtxB (6.5 nm (27)) and albumin (6.8 nm (28)). Co-localisation of internalised dendrimer with immunolabelled compartments was not performed due to fixation-associated artefacts that can misrepresent polymer uptake patterns.

CtxB binds to GM_1_ ganglioside within ordered microdomains termed lipid rafts and the toxin B subunit specifically serves to deliver the catalytic A subunit of the holotoxin to the endoplasmic reticulum and subsequently to the cytosol (29). Demonstrable uptake and trafficking of CtxB within primary rat alveolar epithelia indicates Cav-1 functionality in the pulmonary epithelial barrier. To complement this, BSA was used as a probe for non-specific fluid-phase endocytosis as it results in considerably less background fluorescence on the glass coverslips after short incubation periods compared to, for example dextrans. This can be particularly challenging when imaging ATI-like cells due to the highly attenuated cell periphery (30). The internalisation of albumin has been linked to the gp60 (albondin) receptor-mediated uptake of proteins in lung alveolar epithelial cells (31) although this has not been widely confirmed.

Lung ATI cells represent 95% of the lung absorptive surface area and express caveolin-1 (Cav-1) as a phenotypic biochemical marker. Cav-1 (32,33) and macromolecular trafficking via caveolae has been targeted for enhanced delivery of nanoparticles and macromolecules (34). A detailed investigation of the trafficking mechanisms of PAMAMs in lung epithelia was beyond the scope of this study. Nonetheless, these data provide a novel insight into the endocytic capacity of ATI cells and lead us to conclude that the endocytic machinery of alveolar epithelia, including Cav-1 positive vesicles (35,36), fulfil a role in the uptake and intracellular trafficking of macromolecules such as PAMAM dendrimers at the air-blood barrier, as described previously (37) for other cellular barriers. The contribution of endocytic/transcytotic mechanisms to the bulk transport of polymers across epithelial barriers is extremely challenging to deconvolute. The use of chemical and/or genetic knockdown techniques inevitably leads to the modulation of the paracellular transport route, which is most likely the predominant transport pathway for unmodified polymeric carriers displaying sub-10 nm dimensions.

We next employed the IPRL model to study transpulmonary PAMAM transport. This model gives insight into the complex parallel dispositional barriers to absorption from the airways within an intact lung architecture without the confounding whole body distribution and elimination processes such as mucociliary clearance (MCC). The absorption kinetics of PAMAMs and control FD probes displayed a reciprocal dependency between transport and solute size, consistent with a restricted diffusional process. More importantly, anionic PAMAM dendrimers displayed modest lung transport kinetics cf. similarly sized FD solutes. We have previously demonstrated (38,39), using a range of small molecule probes, that the IPRL model discriminates between rapidly absorbed lipophilic compounds such as digoxin (absorption t_1/2_, 6 min) from polar, paracellular probes such as mannitol (absorption t_1/2_, 63 min). The range of absorption half-lives recorded for PAMAMs here spanned 100–600 min, illustrating the prolonged transpulmonary absorption cf. smaller solutes. A rate of polymer clearance from the lung airways that is inversely proportional to molecular weight is, of course, not unique, with comparatively recent studies reporting similar profiles with polylysine dendrimers (40), polyethylene glycol polymers (0.5 to 20 kDa) (41) and hyaluronic acid polymers (7 to 741 kDa) (42). In the case of dendrimer administration to the lung Ryan *et al*. (40) recently reported a relative lung bioavailability of 20–30% for pegylated polylysine dendrimers in the range of 11-22 kDa (3.0–5.7 nm) after *in vivo* intratracheal instillation. da Rocha and colleagues (43,44) administered to mice G3 PAMAM and its pegylated derivatives by pharyngeal aspiration and reported absorption kinetics that were significantly faster than those reported here i.e. absorption half-lives in the range of 120 min. Direct comparison of our data with others is precluded by contrasting approaches such as the use of different PAMAM types, deposition in the central lung *in vivo* cf. peripheral lung ex vivo, as well as dispositional disparities, including the presence of mucociliary clearance *in vivo*, which permits a contribution to bioavailability by gastrointestinal absorption. It should be highlighted that the tracheobronchial circulation is not perfused in the IPRL model and the forced solution instillation delivers >90% of the instilled dose into the peripheral lung regions, with the remaining fraction largely confined to the dosing apparatus (10). In combination, these two key features of our IPRL model allow us to interpret the appearance of solutes in the recirculating pulmonary perfusate as a direct consequence of absorption across the deep lung epithelium, with negligible impact of MCC. In contrast to other earlier works in other mucosal models (45) our data indicate that deep lung delivery of anionic PAMAMs is unlikely to enhance drug absorption kinetics. Hubbard *et al*. (46) concluded that PAMAMs exhibit only modest transepithelial transport across intact human intestinal epithelial sheets and that, similar to our results, transport rate is overestimated by in vitro (Caco-2) monolayers. We would therefore advise that caution should be exercised when interpreting only in vitro permeability profiles of dendrimeric carriers for lung delivery. Our observation does not exclude the opportunity to exploit PAMAMs for controlled release of low molecular weight drugs into or across the lung epithelia as previously demonstrated with PEG polymers (47).

The biocompatibility of inhalation delivery platforms is a critical consideration in the future development of nanomedicines. The decreased permeability observed in both Calu-3 and IPRL studies indicates that PAMAM 5.5 reduces the concentration of diffusible F-Na at the epithelial surface. Bonizzoni *et al*. (48) recently showed that cationic PAMAM dendrimers reversibly interact with, and induce aggregation of, the structurally related fluorescein dye, 5,6-carboxyfluorescein leading to dye sequestration. Anionic dendrimers would not be expected to drive such an exact binding event. More likely could be the interaction between fluorescein and the secondary and tertiary amine groups within the dendrimer core.

The capacity for lung-instilled solutes to cause epithelial barrier disruption was reported previously. Intra-tracheal instillation of cationic PAMAM G3 has been associated with acute lung injury (49). Similarly, we recently showed that instillation of select cationic, amphipathic peptides into the IPRL causes dose-dependent increases in the permeability to mannitol (12). Our data complement a number of in vitro cytotoxicity studies (50,51) which conclude that anionic PAMAMs offer greatly reduced potential to cause cellular damage compared to cationic dendrimers.

We reported above that at higher doses both the PAMAM3.5 and 5.5 species showed a reduced transport rate constant (K_*ini*_) and extent (%) of absorption within the IPRL. Intriguingly, transport of the similarly sized FD20 polymer was identical across a 10-fold dose range, indicating that dose-dependent phenomenon for PAMAM3.5 and 5.5 is not a generalised issue within the IPRL model but rather a feature of the PAMAM polymer species. A technical point is that size exclusion chromatography performed as previously described (10) upon all labelled PAMAM and FD dosing solutions and upon the respective 60 min IPRL perfusate samples (data not shown), confirmed the integrity of all fluorescent conjugates and as such the validity of the kinetic transport interpretation. We wondered if dose-dependent PAMAM transport may be due to PAMAM aggregation within the lung lining fluids. Using SANS analysis of PAMAMs in a relatively simple model of the lung lining fluid we observed that PAMAM generations 3.5 and 5.5 do not aggregate or adsorb to DPPC vesicles. The 20 Å increase in the bilayer thickness of d-DPPC vesicles in the presence of 4 mg/ml BSA or PAMAM G5.5 + 4 mg/ml BSA is not interpretable as a binding or membrane insertion. Using the simplest conceptual model of albumin or PAMAM binding with DPPC vesicles, we predict that the average DPPC vesicle radii would increase by at least one BSA/PAMAM molecular diameter (40–60 Å). The pulmonary (alveolar) epithelial surface is covered with surfactant composed predominantly of lipid (90%) and with the remainder protein. Dipalmitoylphosphatidylcholine (DPPC) is the major lipid component, comprising 30–60% of the total lipid surfactant content (52). Interactions between PAMAM dendrimers and model lipid membranes have been previously investigated using a range of biophysical methods to examine membrane interaction and cytotoxicity (53–55). The majority of published studies on dendrimer-membrane interactions have focussed upon cationic PAMAM dendrimers. However, of particular relevance to our data Lombardo *et al*. (56) recently observed DPPC alkyl chain disruption by PAMAM 2.5, which was mirrored by increases in vesicle zeta potential and modulated SAXS spectra. The technique of co-dissolution of PAMAMs with DPPC prior to liposome extrusion, as used by Lombardo *et al*., is likely to increase the probability of intermolecular interactions between the components and the entrapment of dendrimer within the interfacial lipid region. This is highly unlikely in our system when PAMAM were mixed with colloidally stable DPPC vesicles. Here we have excluded PAMAM aggregation with DPPC or albumin fluid components. The possibility remains that PAMAM aggregation or sequestration with other lung luminal components, e.g. complex formation with innate cell membranes (57), innate defence proteins, other serum proteins or surfactant proteins, could explain the dose-dependent transport kinetics.

## Conclusion

Transport of PAMAM 3.5 and 5.5 across *in-vitro* Calu-3 monolayers was faster than predicted by solutes of comparable molecular dimensions but slower than that reported by others in different epithelial monolayer systems e.g. intestinal models. The expression and functionality of endocytic uptake pathways, including caveolae, in primary alveolar epithelial cells were confirmed for the uptake of PAMAM dendrimers as well as model macromolecular probes. In an ex-vivo IPRL model dendrimers did not compromise epithelial barrier integrity, although the airway-to-blood transport kinetics were dose-dependent and restricted compared to solutes of comparable size. Neutron scattering experiments confirmed that PAMAMs do not aggregate in a model lung lining fluid supporting the conclusion that PAMAM self-association is unlikely to be a significant factor in the pulmonary disposition of dendrimer carriers. In summary, anionic PAMAM dendrimers have a favourable lung biocompatibility profile, display rapid uptake into respiratory epithelia and prolonged lung transport kinetics. These findings strongly support the future development of inhaled PAMAM-based drug delivery systems for modified local drug delivery to lung epithelia as well as transpulmonary drug delivery.

## Electronic supplementary material

ESM 1 (PNG 829 kb)

## Abbreviations

BSA: Bovine serum albumin
DPPC: 1,2-dipalmitoyl-sn-glycero-3-phosphocholine
IPRL: Isolated perfused rat lung
MRI: Magnetic resonance imaging
PAMAM: Polyamidoamine
