# Supplementary figures and images for: Endocytic Uptake, Transport and Macromolecular Interactions of Anionic PAMAM Dendrimers within Lung Tissue
Source: Pharm Res. 2017 Jun 14;34(12):2517–31. doi: 10.1007/s11095-017-2190-7 (PMC5736778; doi:10.1007/s11095-017-2190-7)

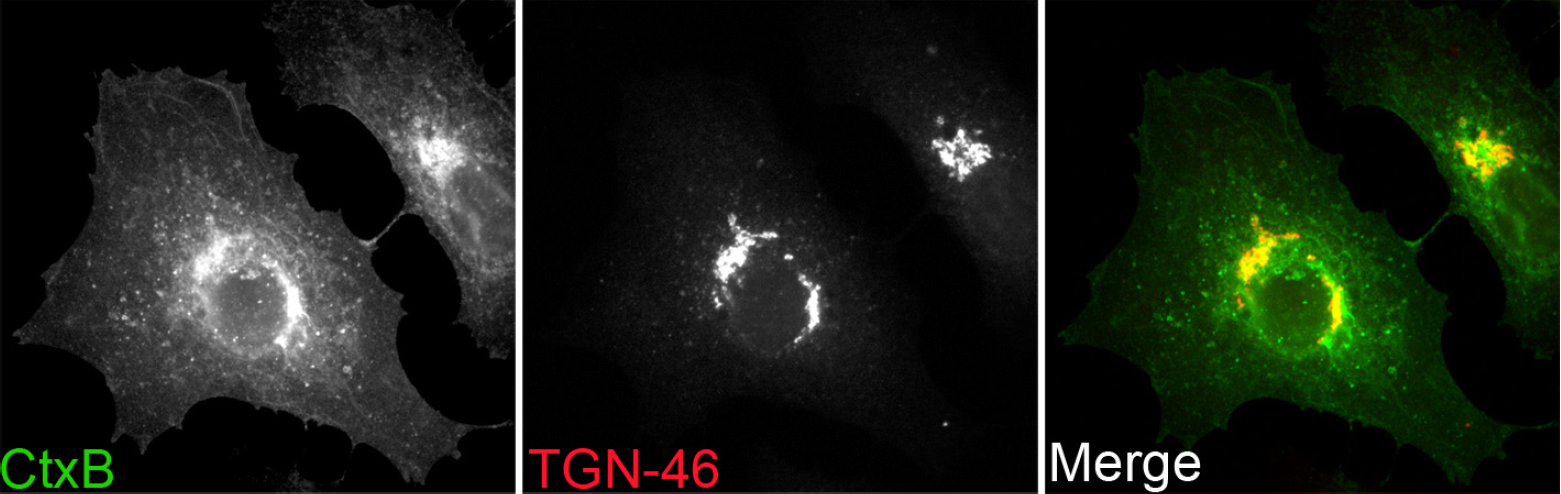

Supplement: Supplementary file 1 — (PNG 829 kb) [file 11095_2017_2190_MOESM1_ESM.png]
